# Analysis of the Impact of Isoquinoline Alkaloids, Derived from* Macleaya cordata* Extract, on the Development and Innate Immune Response in Swine and Poultry

**DOI:** 10.1155/2016/1352146

**Published:** 2016-11-30

**Authors:** Hengjia Ni, Yordan Martínez, Guiping Guan, Román Rodríguez, Dairon Más, Hanhui Peng, Manuel Valdivié Navarro, Gang Liu

**Affiliations:** ^1^Key Laboratory of Agro-Ecological Processes in Subtropical Region, Institute of Subtropical Agriculture, Chinese Academy of Sciences, Hunan Provincial Engineering Research Center of Healthy Livestock, Scientific Observing and Experimental Station of Animal Nutrition and Feed Science in South-Central, Ministry of Agriculture, Hunan Co-Innovation Center of Animal Production Safety, Hunan 410125, China; ^2^College of Bioscience and Biotechnology, Hunan Agricultural University, Changsha, Hunan 410128, China; ^3^Centro de Estudios de Producción Animal, Universidad de Granma, Apartado Postal 21, Bayamo, 85100 Granma, Cuba; ^4^Instituto de Ciencia Animal, Apartado Postal 24, San José de Las Lajas, Mayabeque, Cuba

## Abstract

Medicinal extract has been chronicled extensively in traditional Chinese medicine. Isoquinoline alkaloids, extract of* Macleaya cordata* (Willd.) R. Br., have been used as feed additive in both swine and poultry. Dietary supplementation with isoquinoline alkaloids increases feed intake and weight gain. In addition, recent researches have demonstrated that isoquinoline alkaloids can regulate metabolic processes, innate immune system, and digestive functioning in animals. This review summarizes the latest scientific researches on isoquinoline alkaloids which are extracted from* Macleaya cordata* (Willd.) R. Br. This review specifically focuses on its role as a feed supplement and its associated impact on growth performance and innate immune system, as well as its capacity to act as a substitute for oral antibiotics.

## 1. Introduction

*Macleaya cordata* (Willd.) R. Br., also known as* Bocconia cordata* or plume poppy, belongs to the Papaveraceae family. It is an herbaceous perennial plant, ubiquitously dispersed in central and southeastern China. It is also found in the regions where the parasitic disease (schistosomiasis) is prevalent [1, 2].

*Macleaya cordata* (Willd.) R. Br. contains a number of important alkaloids, which include sanguinarine (SG), dihydroderivative (DHSG), chelerythrine (CH), protopine (PR), allocryptopine (AL), and phenolic acids [3, 4]. A small amount of other isoquinoline alkaloids have also been traced in this plant, such as chelirubine, macarpine, sanguidimerine, chelidimerine, homochelidonine, cryptopine, berberine, coptisine, chelilutine, bocconarborine A, bocconarborine B, oxysanguinarine, norsanguinarine, angoline, bocconoline, 6-ethoxychelerythrine, 6-ethoxysanguinarine, protopine-N-oxide, 6-methoxydihydrosanguinarine, 6-acetonyl-dihyrochelerythrine, and 6-acetonyl-dihydrosanguinarine [3].

*Macleaya cordata* (Willd.) R. Br. grow above the ground and have been used as traditional Chinese medicine for a long time. They are utilized for specific purposes, such as pain relief, modification of the immune system, and reduction of inflammation. The capacity to suppress the proliferation of bacteria, fungi, and viruses [5] has been ascribed to the quaternary benzo[c]phenanthridine alkaloids (QBA), SG and CH [2, 6, 7]. Furthermore, its positive effects on health are evidenced by its ability to inhibit the growth of microorganisms, to block the release or action of adrenaline at nerve endings, to decrease the excitation of sympathetic nervous system, to prevent from fungal infections, and to be used in the treatment of cancer. It also can act as an antiseptic compound, a pesticide against molluscs, and an agent to destroy plant-parasitic nematode worms [2, 8–11].

More recently, food supplements derived from plants have been fed to farm animals. Gradually, they have evoked attention as a substitute to antibiotic growth promoters [12]. This is attributed to the fact that these plants and their extracts are natural substances. They are found to be beneficial in improving growth performance, digestive function, and the absorption of nutrients. They are also helpful in improving the ability of anti-infection and reducing the incidence of diarrhea [12–18].

Based on these properties,* Macleaya cordata* (Willd.) R. Br. showed up in the European Food Safety Authority (EFSA) database. It is employed as a feed additive in intensive livestock farming in an effort to elevate daily food consumption and growth performance [19–24]. According to Mellor [25] and Le Floc'h and Seve [26], sanguinarine can regulate the serotonin synthesis by employing tryptophan and finally lead to improvement in feed intake [20]. However, more studies are required to investigate the effects of extract of* Macleaya cordata* (Willd.) R. Br. on pigs fed with tryptophan-deficient diet [27, 28].

Some investigations have revealed that dietary supplementation with isoquinoline alkaloids reduced the diarrhea and improved gut health, immune system, and digestive function in nonruminant mammals [17, 18, 22, 29]. Therefore, the primary goal of this review was to discuss the impact of isoquinoline alkaloids, derived from extract of* Macleaya cordata* (Willd.) R. Br., on the growth and immune system in swine and poultry.

## 2. The Impact of Isoquinoline Alkaloids, Derived from Extract of* Macleaya cordata* (Willd.) R. Br., on the Growth of Animals

### 2.1. Swine

Phytobiotics can be defined as plant derived products added to feed in order to improve performance. It can be obtained through combining a large array of herbal-based products [30]. A number of researchers have claimed that some plants, as well as their extracts, are able to increase appetite and activate endogenous secretions of enzymes and hormones [13, 17, 31]. In the case of treatment of diseases, they have also been found to have the capacity to destroy microorganisms and parasitic worms in nonruminant animals. Moreover, they are able to retard the growth and reproduction of coccidian parasites [30].

Evidence is available from numerous studies to substantiate that adding phytochemical ingredients to the diet of pigs had beneficial outcomes, particularly in the treatment against growth retardation and disease. As antimicrobial agents, their efficacy is influenced by the concentration of additives and the pH in the animal's intestine [32]. Numerous researches revealed that phytochemical ingredients can reduce coliform bacteria in gastrointestinal tract (GIT) and decrease the diarrheal frequency or mortality rates among young pigs. Phytochemical additives also play an important role in deterring diarrhea and oedema in piglets during the weaning process [12].

Growth performance, as Kong et al. [13] and Jobgen et al. [33] stated, is a complicated progress involving the delicate interaction between metabolism and catabolism. But we may infer the potential physiological or biochemical effect of food additives on the animals through the investigation on the metabolites. For example, the metabolic properties of intracellular protein and the rate of fat deposits are valuable references for the determination of appropriate glucose and amino acid usage. It is no doubt that the metabolic processes are also modulated by hormones and other elements. Both antibiotics and extract of* Macleaya cordata* (Willd.) R. Br. can be used as growth promoters. When a comparative analysis was undertaken between them, the extract demonstrated similar effect as antibiotics on the intestinal health and growth performance [17, 29].

Using the extracts of* Macleaya cordata* (Willd.) R. Br. as feed additives at the concentration ranging from 15 to 50 mg/kg, increased weight gain was found [17, 29]. This outcome has been attributed to the positive influence of internal and external factors on animal production, particularly due to their antimicrobial properties and their capacity to modify immune system and the reduction of inflammation [34]. A number of bacteria located in the mouth cavity of humans were identified to have antimicrobial qualities. Some of these bacteria were classified among the species frequently situated in the GIT of swine [35, 36]. Feeding sanguinarine at minimal inhibitory concentration showed similar effect on bacteria. This may indicate that dietary supplements militate against the rapid multiplication of pathogen bacteria located in the GIT, which in turn impacts upon developmental progress.

From a scientific perspective, the primary contentious issue is about the effect of isoquinoline alkaloids from* Macleaya cordata* (Willd.) R. Br. on feed intake in farming animals. Some studies claimed that sanguinarine additives had no impact on feed consumption [28, 37]. Conversely, other researchers [25, 26] subscribed to the belief that sanguinarine could influence feed intake by regulating the pathway for the synthesis of serotonin by using tryptophan. One study showed that sanguinarine led to greater feed intake (increased by 7%) and acquisition of nourishment, compared to those fed with antibiotics [17]. Beneficial effect on nitrogen balance and growth performance was also found when sanguinarine was added to the diet of swine [20].

No toxicity was found when swine and mice ingested the plant* Macleaya cordata *(Willd.) R. Br., let alone its alkaloid extract, because most of the possible contaminants had been removed [35, 38, 39]. Thus, adding the herb or/and its extract into animal feed would not expose the consumer to dangers. Furthermore, no negative impact on health was detected [35].

In addition, the introduction of isoquinoline alkaloids has decreased the prevalence of diarrhea [18]. Typically, diarrhea is associated with rapid multiplication of* Escherichia coli* and other pathogens in the intestine. The abnormal proliferation of bacteria results in the excretion of water and electrolytes through the semifluid feces and urine [13]. Isoquinoline alkaloids in the extract were found to suppress or destroy these microorganisms, as well as modulating vital functions, such as peristalsis and the pH of intestines [12].

Research conducted by Walker [8] and Newton et al. [9] confirmed that sanguinarine acts as an antimicrobial agent. They found that diet supplemented with sanguinarine had the potential to facilitate the establishment of beneficial bacteria in the GIT of swine, as well as the reinforcement of competitive exclusion principle by inhibiting the colonization of pathogenic bacteria. In addition, sanguinarine reduced the water loss in the epithelial cells of the intestines and/or enhanced the intestinal function in the absorption of water and nutrients [13]. The escalation of metabolic rates of biomolecules and the antioxidant capabilities in the small intestinal mucosa appeared to generate these effects [40].

One study demonstrated that the introduction of feed additives in the form of* Macleaya cordata* extract, containing isoquinoline alkaloids, increased the serum amino acids in swine [41]. And isoquinoline alkaloids can strengthen the capacity to assimilate and absorb ingested protein and AA. In addition, it is likely that this compound modulates the metabolism process in relation to the absorption of nutrients through signal transduction pathways. Nutrients augmentation in portal vein (and specifically AA) which derives from the small intestine may be adequate to stimulate tissue protein synthesis in animals, which has benefit impacts on the growth development [40, 42].

A correlation was found between feed additives and the enhanced movement of amino acids, leading to growth improvement. Greater volumes of essential amino acids, such as lysine, shield the intestine from pathogens and perform a crucial function in calcium absorption. They are also helpful in the preparation of muscle protein, hormones, enzymes, and antibodies [43, 44]. For example, arginine participates in various pathways, including the production of proteins, nitric oxide, polyamines, and creatine [45]. Methionine is another key intermediate in the biosynthesis of proteins and phospholipids. In addition, this amino acid, along with choline, contributes to transfer fat, thus decreasing the fat levels in liver. Methionine also has antioxidant property, and it comprises the element sulfur, which assists in neutralizing free radicals which emerge as a consequence of the diverse components of metabolism [40].

### 2.2. Poultry

Antibiotics as growth promoters have been withdrawn from the feedstuffs of poultry in most regions of the world. Therefore, an increasing demand for the exploration of other possible options is arising to sustain growth development. It is also important to ensure that beneficial microorganisms are predominant in the intestine to specifically prevent the proliferation of pathogenic bacteria. A number of plant additives have been extensively utilized to sustain or enhance the growth performance in poultry [46]. In addition, herb extracts may boost their immune system and decrease blood cholesterol levels [47].

Research has demonstrated that isoquinoline alkaloids prevent the spread of specific bacteria that generate gastrointestinal distress [48]. They also improve appetite and the growth performance [20]. In the case of broiler chickens and maturing turkeys, the recommended dose of* Macleaya cordata* in diet is 20 to 50 ppm [49].

Variations have emerged in the studies conducted to measure the impact of isoquinoline alkaloids on broiler chickens. One study found that when chickens (Cobb × Cobb, male) ingested isoquinoline alkaloids at the dose of 25 and 50 ppm, the body mass and feed conversion rate increased [36]. Notwithstanding this, another research focusing on maturing Ross 308 chickens did not reach a similar conclusion. It found that isoquinoline alkaloids administration at 20 mg/kg failed to influence the growth development and the protein utilization in the poultry [50].

Nevertheless, the introduction of isoquinoline alkaloids into the diet has been claimed to have impact on gastrointestinal performance and the fermentation metabolic process in terminal GIT. It has also been confirmed that isoquinoline alkaloids influence the gastrointestinal movements [51]. Jankowski et al. [52] reported that adding* Macleaya cordata* compounds to the diet of broiler chickens could decrease inordinate fermentation in the caecum without disturbing the pH levels in this area, leading to the enhancement of growth performance.

## 3. The Effects of Isoquinoline Alkaloids, Derived from* Macleaya cordata* Extract, on the Innate Immune Response

### 3.1. Swine

Young pigs, weaned prior to the usual period (ranging from 15 to 28 days old), were subjected to situational tension and nutritional deficits, resulting in the rapid multiplication of intestinal disease-inducing bacteria (e.g.,* Escherichia coli*). In addition to growth retardation, it led to higher morbidity and mortality rates [13, 53]. This demonstrates that the innate immune system operating in young animals influences their performance levels, as well as their response to stimuli.

The innate immune system is an important subsystem of the overall immune system that comprises the cells and mechanisms that defend the host from infection by another organism. This implies that, within this immune system, cells identify and react to pathogen in a nonspecific manner. In contrast to the acquired immune system, it cannot endow immunity over a prolonged time period or defend its host. This innate immune system offers instantaneous protection from disease [54].

Throughout this phase, instantaneous defense is ensured by stimulating the inherent immune cells macrophages, as well as other cells such as the dendritic, polymorphonuclear, and epithelial. This occurs as a result of various toll-like receptors which identify crucial molecules on the outer layer of the bacteria [55]. Neutrophilic granulocytes consist of lysozyme, in primary as well as secondary granules. Its key role is to defend against pathogens and various foreign bodies surrounding the host [55]. This process is fully accomplished through phagocytosis and digesta. Therefore, the ingestion of isoquinoline alkaloids contained in extract of* Macleaya cordata* (Willd.) R. Br. was considered to be essential throughout crucial developmental phases, especially while the species is primarily dependent on intrinsic immunity [35]. The compound activates phagocytes, hence stimulating the organism's defense mechanisms [29].

Intestinal barrier systems are rigorously managed by a meticulously construed epithelial junctional complex, commonly known as the “the tight junction” [56]. It comprises a number of different proteins, which include a transmembrane protein called occludin [57], various derivatives of the claudin group, a junctional adhesion molecule [58], and several linker proteins, for example, ZO-1. Three of the most crucial and beneficial proteins are occludin, ZO-1, and claudin-1, as they play an important role in the control of the tight junctions [59]. In connecting the C-terminal selections of *β*-actin and occludin [18], ZO-1 is a helpful linker protein in the tight junction.

Research has found that the ingestion of extract of* Macleaya cordata* (Willd.) R. Br. can increase the expression of ZO-1 and claudin-1. Thus, it is helpful in preventing allergenic and toxic matter entering the intestines and reducing risks [60]. This is indicates that the use of extract of* Macleaya cordata *(Willd.) R. Br. as a feed additive can promote intestinal mucosal growth and improve defense systems [18].

Recent research conducted by Kantas et al. [29] revealed that the introduction of alkaloids into the feedstuffs reduced the haptoglobin level in swine. This protein is found in blood plasma. It usually binds free hemoglobin and forms the hemoglobin-haptoglobin complex. Then the complex is withdrawn from circulation by the liver, whereupon it participated in a catabolic process in hepatic parenchymal cells. This study also demonstrated that dietary supplementation with alkaloids reduced the level of serum amyloid A (SAA). These proteins are a group of apolipoproteins produced in reaction to cytokines, which are stimulated by monocytes or macrophages. They are closely associated with inherent immunity. The long-established belief was that SAA performed a crucial function in relation to the biological mechanisms that lead to disease in amyloid A-type amyloidosis [61]. Hence, dietary supplementation with isoquinoline alkaloids extracted from* Macleaya cordata* (Willd.) R. Br. boosts the immune system and regulates metabolic process and finally promotes growth and development in swine.

### 3.2. Poultry

Antibiotics are usually replaced with probiotic, organic acids, and herbal extracts in poultry diet. Thus, it was necessary to clearly define the function of these compounds. Given these concerns, Yakhkeshi et al. [62] conducted a comparative analysis to determine the impact of herbal extracts, probiotics, organic acid, and antibiotics on the serum lipids, immune response, intestinal structures, and microbial population in broilers. No substantial variations were found in weight gain and feed conversion ratio when broilers were aged 1–14 and 14–28 days.

Furthermore, this study found that the alkaloids contained in these compounds substantially enhanced intestinal health and the absorption of nutrients. Unlike other interventions, the addition of sanguinarine to the diet resulted in a substantial rise in the heterophils to lymphocyte ratio (H/L). Evidence has shown that herbal extracts improve antibody titration against sheep red blood cells (SRBC). Studies have also demonstrated that herbal extracts trigger the immune system by boosting vitamin C levels. It has been recognized that isoquinoline alkaloids have the capacity to adjust or regulate immune functions [14]. In addition, this medicinal compound can activate phagocytosis, hence prompting defensive reactions by the host [63].

The introduction of isoquinoline alkaloids to the diet of broilers has been shown to considerably reduce the villus height of intestine and the depth of glandular layer [52]. But Vieira et al.[36] found no significant differences in villus height and crypt depth in broilers fed with and without sanguinarine. As far as we know, villus height and intestinal surface area are positively correlative to nutrients absorption and health in animals [64]. It was noted that cells situated in the villi (such as inflammatory cells or enterocytes) are also important when health problems exist. Typically, a greater volume of goblet and immunocyte are not directly correlated with nutrient absorption. But they were found to decrease absorption levels due to enhanced intestinal viscosity and the rate of passage of feeds.

Research undertaken by Pickler et al. [64] showed that the decrease of CD3 cells (this indicator relates to T lymphocytes cells) was detected in the duodenum, jejunum, and ileum of broilers fed with sanguinarine. More goblet cells were noted in the duodenum and ileum in control group compared with the group fed with sanguinarine. Sanguinarine was also found to alleviate the injury of mucosa, suggesting that it would be helpful to prevent enterobacterial infection.

This review has highlighted the idea that dietary supplementation with isoquinoline alkaloids, the extract of* Macleaya cordata* (Willd.) R. Br., is beneficial to swine and poultry. This compound increases feed consumption, body mass, and weight gain, as well as the concentration of serum amino acids. It boosts the innate immune system by regulating phagocytes, haptoglobin, and amyloid A. In addition, it promotes effective gastrointestinal movements, as well as carrying out an important intestinal barrier function by action of ZO-1 protein and claudin-1.
